# A Mobile Phone App to Support Young People in Making Shared Decisions in Therapy (Power Up): Study Protocol

**DOI:** 10.2196/resprot.7694

**Published:** 2017-10-30

**Authors:** Louise Chapman, Julian Edbrooke-Childs, Kate Martin, Helen Webber, Michael P Craven, Chris Hollis, Jessica Deighton, Roslyn Law, Peter Fonagy, Miranda Wolpert

**Affiliations:** ^1^ Evidence Based Practice Unit, University College London and the Anna Freud National Centre for Children and Familes London United Kingdom; ^2^ Common Room Consulting Ltd London United Kingdom; ^3^ Create Marketing Ltd London United Kingdom; ^4^ NIHR MindTech Healthcare Technology Co-operative Institute of Mental Health University of Nottingham Innovation Park Nottingham United Kingdom; ^5^ Bioengineering Research Group Faculty of Engineering University of Nottingham Nottingham United Kingdom; ^6^ Division of Psychiatry and Applied Psychology University of Nottingham Nottingham United Kingdom; ^7^ University College London and the Anna Freud National Centre for Children and Familes London United Kingdom

**Keywords:** shared decision making, child and adolescent mental health services, mHealth app, feasibility trial

## Abstract

**Background:**

Evidence suggests that young people want to be active participants in their care and involved in decisions about their treatment. However, there is a lack of digital shared decision-making tools available to support young people in child and adolescent mental health services (CAMHS).

**Objective:**

The primary aim of this paper is to present the protocol of a feasibility trial for Power Up, a mobile phone app to empower young people in CAMHS to make their voices heard and participate in decisions around their care.

**Methods:**

In the development phase, 30 young people, parents, and clinicians will take part in interviews and focus groups to elicit opinions on an early version of the app. In the feasibility testing phase, 60 young people from across 7 to 10 London CAMHS sites will take part in a trial looking at the feasibility and acceptability of measuring the impact of Power Up on shared decision making.

**Results:**

Data collection for the development phase ended in December 2016. Data collection for the feasibility testing phase will end in December 2017.

**Conclusions:**

Findings will inform the planning of a cluster controlled trial and contribute to the development and implementation of a shared decision-making app to be integrated into CAMHS.

**Trial Registration:**

ISRCTN77194423; http://www.isrctn.com/ISRCTN77194423 (Archived by WebCite at http://www.webcitation.org/6td6MINP0). ClinicalTrials.gov NCT02987608; https://clinicaltrials.gov/ct2/show/NCT02987608 (Archived by WebCite at http://www.webcitation.org/6td6PNBZM)

## Introduction

Policy makers have emphasized the need for shared decision making to become standard practice and for service users to experience “No decision about me without me” across health care settings [1]. These goals are active within child and adolescent mental health services (CAMHS) in the United Kingdom. For instance, the Chief Medical Officer’s 2012 annual report [2] stated that shared decision making is central to the government’s commitment to improving the health outcomes of children and young people with long-term conditions. A key ambition puts “children, young people and their families...at the heart of decision making” [2].

The Health Foundation [3] describes shared decision making as both a philosophy and a process requiring a partnership between patients and professionals. Decisions are made collaboratively about assessments, interventions, and support strategies. Shared decision making is a key element of a broader person-centered care perspective. Person-centered care principles include offering patients personalized care, support, and treatment while enabling and empowering them to build their own capabilities [4]. In line with this, shared decision making places value on a patient’s expertise of their daily lives and difficulties and aims to empower them to communicate their personal values in any decision [5].

There is increasing evidence that ensuring collaborative practice and shared decision making in interventions with those with long-term physical or mental health conditions may contribute to improved self-management and patient activation along with better treatment outcomes [6-9]. Findings from child health settings show that young people have the capacity to be involved in the decisions around their care [10]. However, using shared decision making in the context of CAMHS has unique challenges. This includes the difficulties of initiating complex conversations with highly vulnerable and stressed children [11]. Furthermore, the decisions that arise within a CAMHS context often require ongoing deliberation rather than leading to a clear decision point. They are also likely to involve the multiple perspectives of, for example, families, social workers, and schools. The quality of the therapeutic relationship and availability of resources to support the process were other key factors identified by a recent systematic review [12]. These had an impact on the provision of person-centered care and shared decision making in CAMHS.

In spite of the challenges, findings suggest that once shared decision-making approaches are adopted in CAMHS, clinicians do not report additional risks or adverse events [13]. Conversely, young people’s involvement in decision making may make key decisions more explicit and planned and therefore potentially less risky. Furthermore, child and parent experiences of shared decision making have been shown to be associated with higher levels of symptom improvement [14].

Young people want to be involved in making decisions about their health care and report feeling more in control of their care when they are included in decisions [15]. Parents also feel that their children should be involved in the decision-making process as it may increase their self-esteem and improve their overall welfare [15]. Clinicians appear to experience implementing shared decision making with young people in 3 possible ways: they may feel apprehensive due to perceived risks, they may feel “clunky” if they lack confidence in how to introduce the approach, or they may feel confident once they have found a natural way to incorporate shared decision making into the way they work [13].

Presently, shared decision making is used inconsistently within CAMHS both nationally and internationally. An Australian study asked young people with diagnoses of depressive disorders about their experiences of treatment decision making [16]. Their levels of involvement varied greatly, as did their satisfaction with their levels of involvement. In the National Health Service (NHS), a need to improve patient engagement in decisions around their care has been identified [17]. For instance, young people express an unmet need for access to developmentally appropriate, personally relevant, and accurate information to empower them to make informed decisions about their mental health care [18,19].

Interventions to support shared decision making in mental health services are emerging internationally. Simmons et al [16] developed an online, evidence-based decision aid to support young people facing treatment choices for moderate to severe depression. The aid includes an outline of treatment options, the evidence for each one, and the likelihood of experiencing symptom improvement and side effects plus a space for patients to record what is most important to them.

Interventions are also emerging within CAMHS in the United Kingdom. For example, a range of tools and approaches to support shared decision making has been developed in 4 UK CAMHS [4]. These tools include decision aids such as choice cards and option spreadsheets plus tools to support the identification and expression of feelings, problems, and goals such as a “getting to know you” booklet. The objective of these tools was to change the relationship between young people and their clinicians by encouraging active involvement from young people. Supporting young people to ask questions independently and raise the issues they want to discuss can also facilitate shared decision making [20]. These tools increased collaboration between young people and clinicians, and shared decision making was best facilitated when clinicians were open to changing behaviors and processes and young people were enthusiastic about moving toward a more collaborative relationship with their clinician [4].

Interventions in child mental health settings which include shared decision making have been shown to improve quality of life and satisfaction [21,22]. Online decision aids for young people with depression were found to be acceptable and useful for clinicians and young people [16].

Young people have advised that technology that is engaging, easy to access, informative, empowering, and provides support between sessions would be a particularly useful addition to therapy [23]. The use of technology in mental health care is recommended by the National Institute for Health and Care Excellence 2011 best practice guidance [24], and evidence supports the effectiveness of using mobile phone apps in therapy [25]. Indeed, young people report already using technology as an informal complement to treatment [23].

Encouraging findings have also emerged from an evaluation of tools supporting young people’s mental health through preparing for discussions, mood tracking, and self-management [26]. Young people, parents, and clinicians report feeling positive about integrating the use of certain apps into interventions for young people in mental health settings [27]. However, the content of many youth mental health apps is not based on psychological theories or evidence-based practice [28]. It has been argued that more research is required to better understand how best to integrate digital mental health tools into services [23].

To the best of our knowledge, there are presently no apps designed for and tested in UK CAMHS that support young people to become more actively involved in their care and the decisions surrounding their care. Our research project aims to develop and rigorously test an evidence-based mobile phone app, Power Up, for young people to use alongside CAMHS appointments. This app will provide tools aiming to support young people’s voices in therapy, facilitate a more patient-centered approach, and increase shared decision making. Power Up will enable young people to record their questions, plans, decisions, and diary entries and support young people to decide and remember to whom they could communicate these things. By providing a digital space for young people to prepare what they want to bring to a session, Power Up can support them to actively engage in and direct their therapy.

Study 1 will aim to elicit opinions of young people, parents, and clinicians on an early version of the app, which will inform further developments. A feasibility trial will then be conducted in study 2, which will aim to collect the necessary parameters to plan a cluster controlled effectiveness trial of Power Up.

## Methods

### App Development

New and existing tools that support young person activation, empowerment, and shared decision making will be developed. From existing projects, which the present authors have been involved in, a number of evidence-based paper and online tools to support young people making shared decisions in CAMHS have been developed. These tools aim to provide young people with relevant, accessible information, tailored advice, support with self-management activities, and decision aids [13,29]. Aspects of these tools will be combined with newly developed tools to create the new app, Power Up. The app development process will adhere to best practice guidance for patient decision aids [30], quality criteria for health mobile apps [31,32], and design and evaluation guidelines for mental health technologies [33].

A shared decision-making model for clinical practice was developed by Elwyn et al [34] that identified 3 key steps: choice talk, option talk, and decision talk. During choice and option talk, collaborative decision making is introduced and justified to the patient, and options are then presented. Within decision talk, patients must be encouraged to form and express preferences. The capacity for young people to be involved in such decision talk was identified as a key barrier to shared decision making and person-centered care in CAMHS [12]. Indeed, Elwyn et al [34] identified that some patients are likely to need time and resources to consider what their preferences are and that this deliberation may need to be done outside of the clinical encounter. Power Up therefore aims to provide young people with this space to record their experiences and consider their preferences, consequently increasing their capacity to be involved in decision talk.

### Patient and Public Involvement

Key stakeholders will be heavily involved in the development of Power Up through patient and public involvement (PPI) sessions. A user-centered agile development process will enable feedback from stakeholders to iteratively inform the design of the app. Through these sessions, young people with experience in accessing services, parents, and CAMHS clinicians will be consulted to ensure that the app’s development is stakeholder-led. These groups will be consulted regarding initial ideas proposed for the app’s content, importance and appropriateness of each tool, suitability of the wireframes (images of the functional elements of each screen), design elements, and protocol as it is developed.

PPI will be actively involved in the governance and delivery of the research. A PPI group will be facilitated by the PPI lead (KM) and 2 young advisors with expertise in shared decision making who have been recruited, trained, and supported by the PPI lead in line with best practice guidelines [35]. The group will be actively involved in project governance; cofacilitation of coproduction sessions with local service user groups; design and review of information sheets, informed consent forms, recruitment material, and interview schedules for young people, carers, and therapists; and dissemination of findings. The group will lead coproduction sessions with local service user groups who will act as advisory partners to the project, feeding into management, content development, beta-testing, and feedback for further development. While the project is primarily aimed at supporting shared decision making in young people, it is also crucial to engage with parents and carers. The PPI group will lead coproduction sessions and interviews with parents and carers to ensure their expertise informs project management, content development, and feedback for further development. Coproduction sessions will focus on feedback from young people about the design and refinement of Power Up and making it more intuitive, wording sections for clarity, recruiting for the study, using the app within sessions, and encouraging Power Up to be a personal space for young people.

### Power Up Content and Design

A recent scoping review of approaches to support shared decision making in young people showed that many were not aimed solely at young people; instead, many were aimed at parents [36]. Moreover, those that were aimed at young people tended to miss key areas of shared decision making and tended to be used within or just before appointments rather than being a tool that could help manage difficulties outside of sessions and help young people express their opinions. In addition to this, none were interactive apps; they were instead confined to websites, mostly detailing information. Power Up addresses these gaps by allowing decisions to be tracked, revisited, and reviewed over the course of treatment. Power Up is an app for young people in CAMHS to use independently. The Power Up app will provide young people with tools to use within and between CAMHS sessions. The objective of these tools is to empower young people to be more actively engaged in their care and decisions about their care by providing a space for them to record and prepare what they want to bring to and share in a therapy session.

Users of the Power Up app will not be able to digitally share information that they enter. The value of young people being able to communicate directly with their clinician through the app was considered. It was concluded that, in the context of a feasibility trial, the information governance and data security issues around data sharing through the app could not be sufficiently mitigated at this time. Presently, therefore, Power Up provides a private space for young people to use alongside their CAMHS sessions.

**Figure 1:**
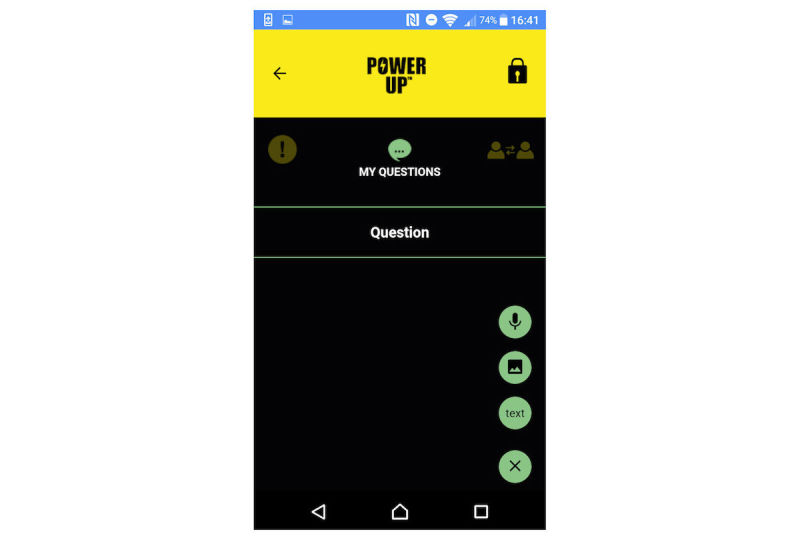
My Questions.

**Figure 2:**
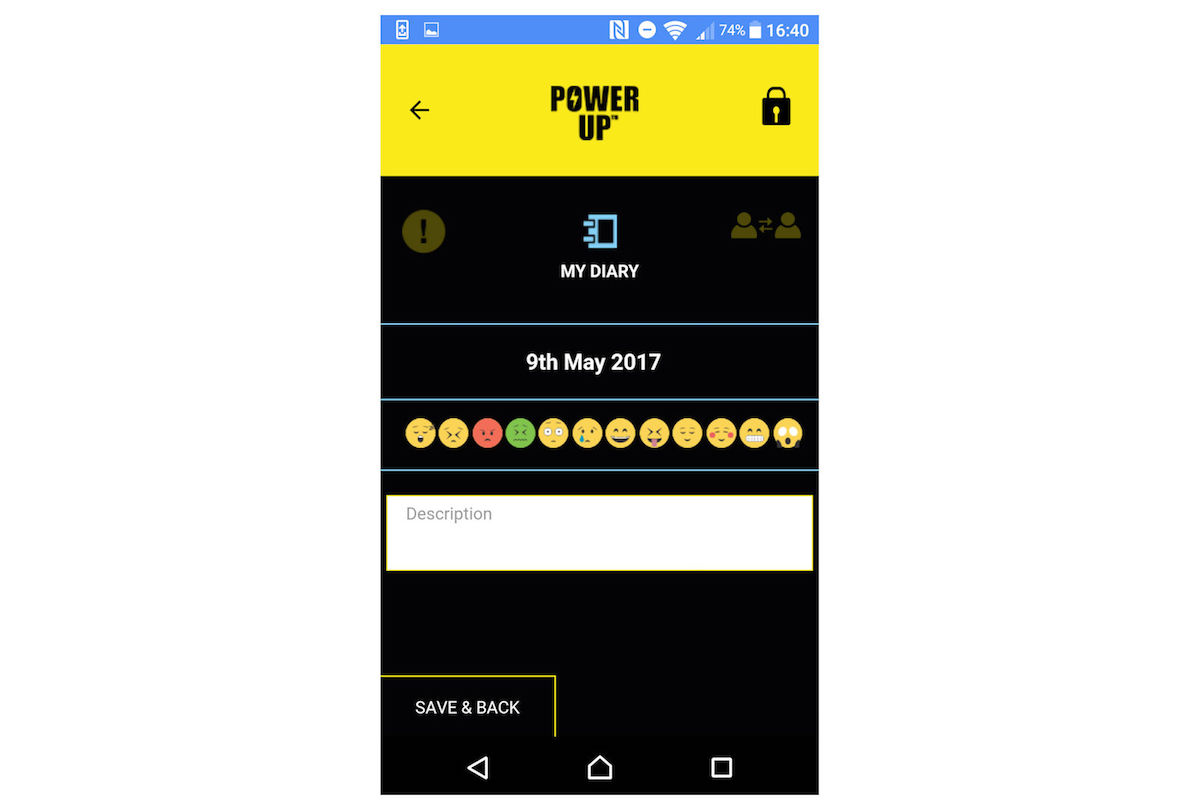
My Diary.

**Figure 3:**
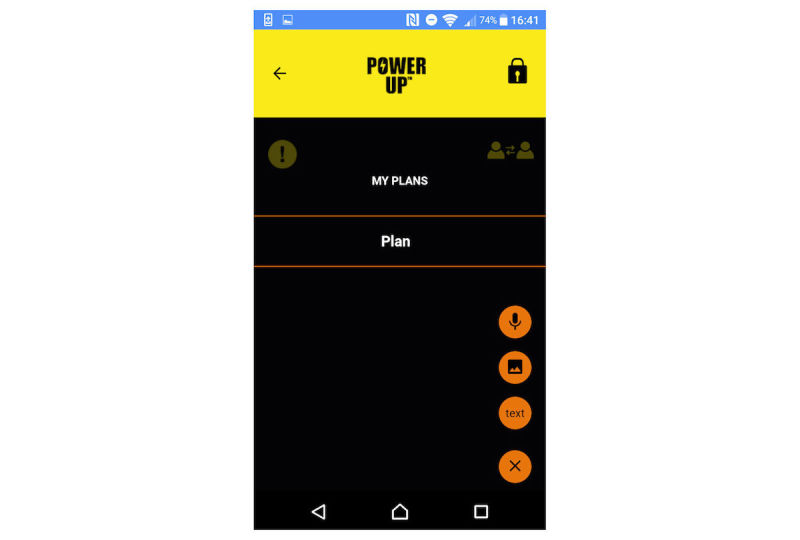
My Plans.

**Figure 4:**
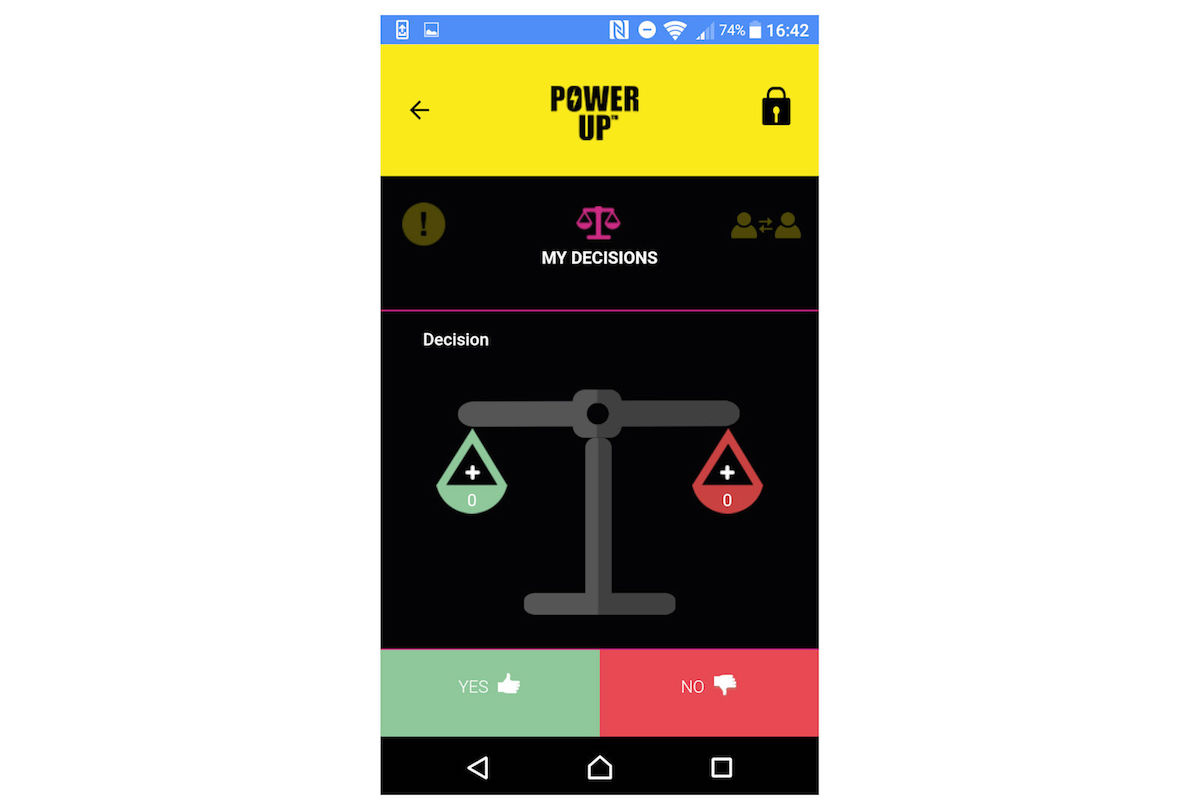
My Decisions.

There are 4 key tools in which young people can use text, audio, video, and photos to create entries. In My Questions (see Figure 1 for screenshot), young people can record questions they would like to ask people in their support network (eg, their CAMHS clinician) and record their responses. In My Diary (Figure 2), young people can record session journals and daily journals, describing their thoughts, feelings, and experiences. In My Plans (Figure 3), young people can record step-by-step plans for achieving goals or tackling difficulties. My Decisions (Figure 4) is a space for young people to enter a decision they want to work through (eg, returning to school). The young person then adds pros (eg, keeping up with school work) and cons (eg, fear of being bullied) for the decision, assigning a weight to the importance of each one.

Additionally, users will record a list of all the people in their support network, including their CAMHS clinician, in My People when they first download the app. As Power Up users add entries to the app, they will be reminded to consider if they want to talk to any of the individuals in My People about their entry.

Finally, the Help and Support tool will signpost young people to other relevant resources giving information and advice. Young people will be able to add their own links and phone numbers to the list.

Our research project will be executed across 2 phases: development and feasibility testing.

### Study One: Development Phase

#### Participants and Design

Using a qualitative design, semistructured interviews and focus groups will be conducted to elicit the opinions of group members concerning the structure and content of a prototype of Power Up. Up to 30 participants will be recruited to study 1: 10 young people, 10 parents and carers, and 10 clinicians. Young people recruited to this study will be aged 11 to 19 years, currently attending sessions in CAMHS, and presenting with emotional difficulties such as anxiety or depression. A clinician will have confirmed that the young person does not have any vulnerability that would make taking part in the study inappropriate to their context. Parents and carers recruited to this study will be over 18 years old and a parent or carer of a child currently attending sessions in CAMHS. All participants will be able to understand English sufficiently to provide informed consent.

#### Procedure and Materials

Participants will be recruited at 3 London CAMHS sites. Contact will be made with various sites well in advance of the study’s start date to ascertain potential interest in the whole project.

##### Clinicians

At the commencement of study 1, the key contact person at each site will set up an initial meeting between clinicians and researchers. During this initial meeting, clinicians will be invited to participate in a focus group or interview. They will be given an information sheet that provides details of the purpose of the study and what their involvement would require. Approximately 10 clinicians will be recruited across the 3 services, and a date will be set between researchers and clinicians by phone or email. They will sign an informed consent form before they take part in the interview or focus group.

##### Young People and Parents

In the initial meeting, clinicians will also receive training on the young person recruitment procedure for stages 1 and 2. Clinicians will select young people currently attending CAMHS by reviewing their patient lists to identify young people who fit the inclusion criteria. The training will highlight that selection bias should be avoided during this process as the views of young people with a range of levels of engagement with therapy are necessary to understand how the app may or may not be used. The number of people screened on the patient lists, number who met the inclusion criteria, and number approached will be recorded.

Through a postal letter, telephone, or face-to-face conversation, young people and their parents or carers will be given information about the study. Young people and their parents or carers will indicate to a member of CAMHS staff or the research team, through a telephone or face-to-face conversation, that they are interested in taking part in the project.

Before the interview or focus group, all participants will read an age-appropriate information sheet outlining the purpose and details of the study. If the young person is aged 15 years or younger, their parent or carer will also be given an information sheet to read. Their parent or carer will sign a parental consent form agreeing for them to take part in the study before the interview or focus group. The young person will sign an assent form indicating they would like to take part. Young people who are aged 16 years or older and their parents or carers will sign their own informed consent forms before they take part. Interviews are expected to last up to an hour.

In the focus groups and interviews, researchers will share the first version of Power Up with participants on a mobile phone. Researchers will talk the participants through the features of the app, and then participants will be given some time to try out this app on a mobile phone provided by the research team. Questions will be asked according to a semistructured topic guide (see Multimedia Appendix 1). Participants will be asked to talk the interviewer through their thoughts as they are looking at the app. Afterwards, questions about the app’s content, usability, usefulness, and design will be asked. At the end of the focus group or interview, participants will be debriefed. They will be given contact details for the researchers should they wish to ask any questions or withdraw their data. Ideally, young people and carers will take part in separate interviews and focus groups; if this is not possible, the researcher will aim to elicit responses from all participants to ensure the voices of young people and carers are heard. Interviews and focus groups will be transcribed verbatim and analyzed using the framework approach to identify themes pertaining to the usefulness, functionality, and design of Power Up to be fed back to the design team and inform subsequent iterations of the app [37]. The analysis will compare and contrast responses from different groups to identify similarities and differences between young people, carers, and clinicians.

### Study Two: Feasibility Testing Phase

#### Participants and Design

In the feasibility testing phase, young people’s experiences of CAMHS while using Power Up will be compared to young people’s experiences of CAMHS without using the app. Study 2 is designed as a feasibility trial using a waitlist control design. A total of 60 young people, who are aged 11 to 19 years old, have recently been referred to CAMHS, and are presenting with emotional difficulties will be recruited to the trial. A clinician will have confirmed that the young person does not have any vulnerability that would make taking part in the study inappropriate to their context. They will also understand English well enough to provide informed consent or assent if they are younger than 16 years old. First, 30 young people will be recruited to the control phase of the trial where they will receive management as usual. Subsequently, 30 different young people will be recruited to the intervention phase of the trial where they will be given Power Up to use alongside management as usual.

#### Procedure

Participants will be recruited in 7 to 10 London CAMHS sites. Clinicians will identify young people who are in their initial sessions in CAMHS. Young people and their parent or carer will be given information about the trial, and they will indicate to a member of CAMHS staff or the research team, through a telephone or face-to-face conversation, that they are interested in taking part in the project. For the control phase and the intervention phase, each service will be required to recruit 5 to 10 young people (for a total of 30 young people across services for each phase). This initial contact by services and subsequent recruitment by researchers will replicate the processes described in study 1.

If the young person is recruited during the control phase, they will meet with a researcher, along with their parent or carer, at a convenient time and place in the early stages of their therapy (up to the third treatment session). The researcher will take informed consent using the same procedures as described in study 1. The young person will then complete a battery of measures that take an estimated 15 minutes to complete. The young person’s parent or carer and clinicians will also complete a short questionnaire each.

Three months later all participants will be recontacted by the researchers and a time will be arranged for them to complete the same battery of measures a second time. Their clinician will also be asked to report the young person’s presenting problems, type of interventions used, number of sessions attended, number of missed appointments, and length of appointments.

Data collection for the intervention phase will replicate the control phase procedures. For those recruited to the intervention phase, the researcher will also help the young person to download Power Up onto their phone and will talk them through its functions after completing the first time measures. The young person will then be able to use Power Up as much as they want throughout their therapy at CAMHS, within and between sessions. In the intervention phase, a total of 10 to 12 young people and 10 to 12 clinicians across services will also take part in a posttrial interview where they will be asked about their experiences of and opinions on using Power Up in CAMHS.

At the end of their involvement in the trial, participants will be debriefed by a researcher. All participants will be offered 5 pounds (US $6.63) travel reimbursement at the end of their involvement in the study. They will be given contact details for the researchers, should they wish to ask any questions or withdraw their data.

### Measures

#### Demographic Characteristics

Participants will be asked to report their age, gender, ethnicity, any disabilities, and first language.

#### Patient Activation Measure–Mental Health

The Patient Activation Measure–Mental Health (PAM-MH) is a patient-reported tool for measuring engagement in mental health care. The 13 items are used to measure patients according to 4 activation levels: skills, knowledge, confidence, and behaviors critical for coping with and managing mental health. Statements such as “When all is said and done, I am the person who is responsible for taking care of my mental health” will be rated using a 5-point response scale ranging from disagree strongly to agree strongly. PAM-MH was adapted from the physical health Patient Activation Measure; an examination of psychometric properties found it appears to be a reliable and valid measure [38]. It has been used in a number of studies with people accessing mental health services (see, for example, Matthias et al [39]). In the proposed feasibility trial, we will collect necessary parameters for planning a full prospective parallel cluster controlled trial to test the effectiveness of Power Up. We consider a minimal clinically important difference to be 55.10 on the PAM-MH in the management as usual arm (indicating they lack confidence to take action to manage their mental health difficulties) versus 67.10 in the Power Up arm (indicating they are able to take action to manage their mental health difficulties) [9].

#### CollaboRATE

CollaboRATE is a 3-item patient-reported shared decision- making measure. The measure assesses the extent to which an explanation of the health issue is given and patient preferences are elicited and integrated. A 10-point response scale from no effort was made to every effort was made is used to measure how much effort was made to “help you understand your health issue,” “listen to the things that matter most to you about your health issues,” and “include what matters most to you in choosing what to do next.” Concurrent validity with other shared decision-making measures and good interrater reliability have been demonstrated in a range of doctor-patient encounters [31]. However, its psychometric properties have yet to be tested in child mental health services. The authors of this study are involved in another study looking at the psychometric properties of CollaboRATE with children and young people.

#### Shared Decision Making Questionnaire

The Shared Decision Making Questionnaire (SDM Q-9) is a 9-item patient-reported shared decision-making questionnaire. Responders rate their agreement with 9 statements related to the decision-making process in healthcare consultations. One minor revision was made to the original version of the SDM Q-9; each item was changed from “my doctor” to “the clinician” to make the items applicable to any professional working with young people in CAMHS. Statements such as “The clinician made it clear that a decision needs to be made,” “The clinician helped me understand all the information,” and “The clinician and I selected a treatment option together” are rated on a 6-point response scale ranging from completely disagree to completely agree. The SDM Q-9 shows a high internal consistency (Cronbach alpha >.9), face validity, and high acceptance [40]. However, its psychometric properties have yet to be tested in child mental health services.

#### Experience of Service Questionnaire

The Experience of Service Questionnaire (ESQ) is a self-completion questionnaire that assesses children and young people’s views of services with respect to accessibility, humanity of care, organization of care, and environment. Responders rate their agreement with 13 statements, such as either “certainly true,” “partly true,” “not true,” or “don’t know.” The ESQ was developed and piloted with CAMHS attendees and has good precision in differentiating satisfaction with care on an individual level in this population [41]. For this study, 4 items of the ESQ will be used as a proxy measure of shared decision making: “I feel that the people who saw me listened to me,” “It was easy to talk to the people who saw me,” “My views and worries were taken seriously,” and “I have been given enough explanation about the help available there.” These items have previously been used as a proxy measure of shared decision making [14].

#### Youth Efficacy/Empowerment Scale–Mental Health

The Youth Efficacy/Empowerment Scale–Mental Health (YES-MH) assesses youth perceptions of efficacy with respect to managing their own mental health condition (self), managing their own services and supports (service), and using their experience and knowledge to help peers and improve service systems (system). The 7 items of the self subscale and 8 items of the service subscale will be used in this research. Responders rate their agreement with statements such as “I feel I can take steps toward the future I want” and “When a service or support is not working for me, I take steps to get it changed” on a 5-point response scale from almost or almost always to never or almost never. Initial analysis of the psychometric properties of this scale when used with 14- to 21-year-olds showed evidence of a clear factor structure and good internal reliability for the self subscale (Cronbach alpha=.88) and the service subscale (Cronbach alpha=.83) [42].

#### Strengths and Difficulties Questionnaire

The Strengths and Difficulties Questionnaire (SDQ) is a self-report behavioral screening questionnaire for children and adolescents measuring symptoms and functioning; 25 items capture 5 subscales, which measure emotional symptoms, conduct problems, hyperactivity/inattention, peer relationship problems, and prosocial behavior. Responders rate their agreement with statements such as “I get very angry and often lose my temper” as either “not true,” “somewhat true,” or “certainly true.” The impact supplement includes 8 items which inquire about chronicity of the difficulties, distress, social impairment, and burden to others. Responses to questions such as “Do the difficulties interfere with your everyday life?” are given using a 4-point response scale ranging from not at all to a great deal. Internal consistency has been judged as satisfactory; the mean Cronbach alpha was calculated as .73 [43]. The SDQ has been used with young people as a clinical assessment tool and in developmental, genetic, social, clinical, and educational research studies.

#### Client Receipt of Services Inventory–Children’s Version

The Client Receipt of Services Inventory–Children’s Version (CSRI) provides information on service utilization as reported by the main carer of the child in the family [44]. Information on background, household circumstances, employment and income, school support, and health service use is recorded regarding the retrospective period of 6 months. For each service type, the number and average duration of contacts is recorded. The CSRI has been previously used in child mental health contexts [45].

#### Dyadic OPTION Scale

This is a 12-item instrument to measure the extent to which patients have been involved in shared decision making from the viewpoint of the clinician. This instrument was adapted from the observer OPTION which underwent extensive psychometric testing. The 2 scales show convergent validity [46], and a systematic review has concluded that the dyadic OPTION scale is the most promising tool for measuring components of shared decision making [47]. Twelve statements, such as “A health problem was identified, where it was made clear that a decision was needed,” “Different options (including the possibility of doing nothing) were discussed,” and “It was made sure that information had been understood,” are measured on a 4-point response scale ranging from strongly agree to strongly disagree.

#### Acceptability Measures

Participants will also be asked to complete a questionnaire about the acceptability of all the above measures.

### Ethics and Informed Consent

Ethical approval has been obtained from Queen Square National Research Ethics Service and the Health Research Authority along with relevant local research governance and site-specific approvals. The trial has been registered with the ISRCTN registry [ISRCTN77194423] and ClinicalTrials.gov [NCT02987608].

Participant information sheets and informed consent forms will be given to all young people (assent forms for young people aged under 16 years), parents or carers, and clinicians. These forms were developed in conjunction with the Core Research Group and the Advisory Group, in particular the PPI coordinator and 2 representatives. The forms will inform participants that participation is entirely voluntary and that it will not impact their care if they decide not to take part. The risks and benefits to the participating people will be addressed and it will be made clear that the data obtained from the study will be confidential and their privacy ensured. Consent forms will also make the participant aware of their right to withdraw at any point during the research.

### Planned Analysis

#### Study One: Development Phase

Focus groups and interviews will be recorded and transcribed verbatim. They will then be analyzed using thematic analysis. Themes will give an understanding of what young people, parents and carers, and clinicians think of the content and format of Power Up. Further developments to the app will be made in response to these themes.

#### Study Two: Feasibility Testing Phase

Descriptive statistics will be used to characterize the participants in terms of sociodemographic profile. The primary outcome measure of the feasibility trial, the standard deviations and intraclass correlation coefficients of the shared decision-making measures, will identify the parameters to enable planning for the subsequent trial. These will be used to calculate the sample size for the future planned cluster controlled trial. In addition, the acceptability of studying Power Up in a cluster controlled trial will be examined using recruitment and retention rates, number of sessions attended, and number of individuals who refuse treatment. The feasibility of studying Power Up will be indicated by the number of patients failing to comply with the full clinical/research protocol and qualitative information obtained from the posttrial interviews with clinicians and young people.

The secondary outcome measures, an initial indication of the impact that Power Up may have on a young person’s clinical outcomes, are ­­­the change in patient activation, empowerment, and SDQ scores pre- and postintervention. Qualitative information regarding the impact of Power Up will also be obtained from the posttrial interviews with clinicians and young people.

The results of this study will clarify the feasibility and acceptability of studying Power Up in a prospective cluster controlled trial. In addition, the results will highlight the possible utility and challenges of implementing Power Up with young people in CAMHS.

## Results

Funding has been secured from the National Institute for Health Research (NIHR)–Central Commissioning Facility to cover the full length of the project. Interviews were completed for study 1 in December 2016. The project is currently in the control phase of the feasibility trial, and 10 CAMHS sites have been recruited to take part in the study. The intervention phase of the feasibility trial commenced in June 2017. It is anticipated that data collection will be completed by December 2017.

## Discussion

The trial and its findings will inform the development and implementation of a shared decision-making app for CAMHS. It will be the first of its kind for young people managing emotional problems in the NHS. This will contribute to the growing use of technology to support children and young people with mental health difficulties.

In addition, the findings will inform the planning of a prospective cluster controlled trial. This larger study will give further evidence of the app’s efficacy in promoting shared decision making in CAMHS while reducing missed appointments and increasing positive outcomes. This will also indicate the potential financial savings the app could have for services. It is hoped that this research and the future trial can work toward putting children, young people, and their families at the heart of decision making about their care.

## Appendix

Multimedia Appendix 1 Topic guides.

## Abbreviations

CAMHS: children and adolescent mental health services
CSRI: Client Receipt of Services Inventory–Children’s Version
ESQ: Experience of Service Questionnaire
NHS: National Health Service
NICE: National Institute for Health and Care Excellence
NIHR: National Institute for Health Research
PAM-MH: Patient Activation Measure–Mental Health
PPI: patient and public involvement
SDM Q-9: Shared Decision Making Questionnaire
SDQ: Strengths and Difficulties Questionnaire
YES-MH: Youth Efficacy/Empowerment Scale–Mental Health

## References

[1] Department of Health (2010). Equity and excellence: liberating the NHS.

[2] Department of Health (2012). Chief Medical Officer's annual report 2012: our children deserve better: prevention pays.

[3] The Health Foundation (2012). Helping people share decision making: a review of evidence considering whether shared decision making is worthwhile.

[4] The Health Foundation 2014.

[5] O'Connor AM, Llewellyn-Thomas HA, Flood AB (2004). Modifying unwarranted variations in health care: shared decision making using patient decision aids. Health Aff (Millwood).

[6] Bodenheimer T, Lorig K, Holman H, Grumbach K (2002). Patient self-management of chronic disease in primary care. JAMA.

[7] Storm M, Edwards A (2013). Models of user involvement in the mental health context: intentions and implementation challenges. Psychiatr Q.

[8] Alegría M, Polo A, Gao S, Santana L, Rothstein D, Jimenez A, Hunter ML, Mendieta F, Oddo V, Normand S (2008). Evaluation of a patient activation and empowerment intervention in mental health care. Med Care.

[9] Hibbard JH, Stockard J, Mahoney ER, Tusler M (2004). Development of the Patient Activation Measure (PAM): conceptualizing and measuring activation in patients and consumers. Health Serv Res.

[10] Alderson P, Sutcliffe K, Curtis K (2006). Children's competence to consent to medical treatment. Hastings Center Report.

[11] Wolpert M, Hoffman J, Abrines N, Feltham A, Baird L, Law D (2012). Closing the gap through changing relationships: final report.

[12] Gondek D, Edbrooke-Childs J, Velikonja T, Chapman L (2016). Facilitators and barriers to person-centred care in child and young people mental health services: a systematic review. Clin Psychol Psychother.

[13] Abrines-Jaume N, Midgley N, Hopkins K, Hoffman J, Martin K, Law D, Wolpert M (2016). A qualitative analysis of implementing shared decision making in Child and Adolescent Mental Health Services in the United Kingdom: stages and facilitators. Clin Child Psychol Psychiatry.

[14] Edbrooke-Childs J, Jacob J, Argent R, Patalay P, Deighton J, Wolpert M (2016). The relationship between child- and parent-reported shared decision making and child-, parent-, and clinician-reported treatment outcome in routinely collected child mental health services data. Clin Child Psychol Psychiatry.

[15] Coyne I (2006). Consultation with children in hospital: children, parents' and nurses' perspectives. J Clin Nurs.

[16] Simmons M, Hetrick S, Jorm A (2011). Experiences of treatment decision making for young people diagnosed with depressive disorders: a qualitative study in primary care and specialist mental health settings. BMC Psychiatry.

[17] Richards N, Coulter A (2007). Is the NHS Becoming More Patient-centred? Trends from the National Surveys of NHS Patients in England 2002-2007.

[18] Day C (2008). Children's and young people's involvement and participation in mental health care. Child Adolesc Ment Health.

[19] Wisdom JP, Clarke GN, Green CA (2006). What teens want: barriers to seeking care for depression. Adm Policy Ment Health.

[20] Simmons M, Rice S, Hetrick S, Bailey A, Parker A (2012). Evidence summary: Shared Decision Making (SDM) for mental health—what is the evidence?. Orygen Youth Health Research Centre.

[21] Asarnow JR, Jaycox LH, Tang L, Duan N, LaBorde AP, Zeledon LR, Anderson M, Murray PJ, Landon C, Rea MM, Wells KB (2009). Long-term benefits of short-term quality improvement interventions for depressed youths in primary care. Am J Psychiatry.

[22] Richardson L, McCauley E, Katon W (2009). Collaborative care for adolescent depression: a pilot study. Gen Hosp Psychiatry.

[23] Montague AE, Varcin KJ, Simmons MB, Parker AG (2015). Putting technology into youth mental health practice: young people's perspectives. SAGE Open.

[24] National Institute for Health and Care Excellence (2011). Common mental health problems: identification and pathways to care.

[25] Gustafson DH, McTavish FM, Chih M, Atwood AK, Johnson RA, Boyle MG, Levy MS, Driscoll H, Chisholm SM, Dillenburg L, Isham A, Shah D (2014). A smartphone application to support recovery from alcoholism: a randomized clinical trial. JAMA Psychiatry.

[26] Simons L, Craven M, Martin J (2015). Learning from the labs, volume 2: evaluating effectiveness.

[27] Simons L, Valentine A, Falconer C, Groom M, Daley D, Craven M (2016). Developing mHealth remote monitoring technology for attention deficit hyperactivity disorder: a qualitative study eliciting user priorities and needs. JMIR mHealth uHealth.

[28] Hides L (2014). Are SMARTapps the future of youth mental health?. Bulletin Aust Psychol Soc.

[29] Wolpert M, Page J, Edbrooke-Childs J (2015). Shared decision-making.

[30] International Patient Decision Aids Standards (2014). IPDAS 2005: criteria for judging the quality of patient decision aids.

[31] Stoyanov SR, Hides L, Kavanagh DJ, Zelenko O, Tjondronegoro D, Mani M (2015). Mobile app rating scale: a new tool for assessing the quality of health mobile apps. JMIR Mhealth Uhealth.

[32] British Standards Institution Health and wellness apps quality criteria code of practice published by BSI.

[33] Doherty G, Coyle DM (2010). Design and evaluation guidelines for mental health technologies. Interact Comput.

[34] Elwyn G, Frosch D, Thomson R, Joseph-Williams N, Lloyd A, Kinnersley P, Cording E, Tomson D, Dodd C, Rollnick S, Edwards A, Barry M (2012). Shared decision making: a model for clinical practice. J Gen Intern Med.

[35] Cartwright J, Kabir T, Simons L (2013). Budgeting for involvement: practical advice on budgeting for actively involving the public in research studies.

[36] Cheng H, Hayes D, Edbrooke-Childs J, Martin K, Chapman L, Wolpert M (2017). Approaches for promoting shared decision making are used in child mental health? A scoping review. Clin Psychol Psychother.

[37] Ritchie J, Spencer L (1993). Qualitative data analysis for applied policy research. Analysing Qualitative Data.

[38] Kelsey J, Abelson-Mitchell N, Skirton H (2007). Perceptions of young people about decision making in the acute healthcare environment. Paediatr Nurs.

[39] Matthias M, Fukui S, Kukla M, Eliacin J, Bonfils K, Firmin R (2014). Consumer and relationship factors associated with shared decision making in mental health consultations. Psychiat Serv.

[40] Kriston L, Scholl I, Hölzel L, Simon D, Loh AM (2010). The 9-item Shared Decision Making Questionnaire (SDM-Q-9): development and psychometric properties in a primary care sample. Patient Educ Couns.

[41] Brown A, Ford T, Deighton J, Wolpert M (2014). Satisfaction in child and adolescent mental health services: translating users' feedback into measurement. Adm Policy Ment Health.

[42] Walker JS, Thorne EK, Powers LE, Gaonkar R (2009). Development of a scale to measure the empowerment of youth consumers of mental health services. J Emot Behav Disord.

[43] Green CA, Perrin NA, Polen MR, Leo MC, Hibbard J HM (2010). Development of the Patient Activation Measure for mental health. Adm Policy Ment Hlth.

[44] Beecham J, Knapp M, Thornicroft G (2001). Costing psychiatric interventions. Measuring Mental Health Needs. 2nd Edition.

[45] Knapp M, Marks I, Wolstenholme J, Beecham J, Astin J, Audini B (1998). Home-based versus hospital-based care for serious mental illness: controlled cost-effectiveness study over four years. Brit J Psychiat.

[46] Melbourne E, Roberts S, Durand M, Newcombe R, Légaré FG (2011). Dyadic OPTION: measuring perceptions of shared decision-making in practice. Pat Educ Couns.

[47] Phillips NM, Street M (2016). A systematic review of reliable and valid tools for the measurement of patient participation in healthcare. BMJ Qual Saf.

