# Supplementary material for: A Mobile Phone App to Support Young People in Making Shared Decisions in Therapy (Power Up): Study Protocol
Source: JMIR Res Protoc. 2017 Oct 30;6(10):e206. doi: 10.2196/resprot.7694 (PMC5684513; doi:10.2196/resprot.7694)
Supplement: Multimedia Appendix 1 [file resprot_v6i10e206_app1.pdf]

Multimedia Appendix 1. Topic guides

Power Up topic guide for clinician interviews and focus groups

Introduction + Aims

- Power Up project – Phase 1 + 2 – timelines
- Description of app
  - Support YP to get their voice heard in CAMHS
  - Space for them to record what's going on for them, and their experience of therapy in their own words (what they enter into the app cannot be digitally shared)
  - Video, audio, text, photo
  - Step by step plans, diary entries, questions, decision tool
  - Record sessions and flag things to talk about in session
  - My library
  - In sessions and between sessions
- Aims of today
  - Some feedback from you on the design and usability of the app
  - Some feedback on how the app could support the therapeutic work you do.
  - Some thoughts on how best to integrate the app into therapy.

Tools (Blind Test first)

- Open up the Power Up app and have a look. Read some of the descriptions of the tools as you open them and start having a play. Add some entries as if you were a user of this app.
- Talk me through your thoughts as you are looking at [Insert Tool name]
  - What do you like/ not like about what Power Up does and how it looks?
  - Is it clear what the tool is for?
  - Is the tool easy to use?

Use of Power Up

- How could Power Up have an impact on young people's experience of CAMHS?
- Can you give me some examples of times in your clinical work that you might want to introduce Power Up to a Young Person?
- Can you give me some examples of times in your clinical work when you would not want to introduce Power Up to a Young Person?
- What adaptions would you have to make to your clinical work to incorporate Power Up?
- How comfortable would you feel introducing a young person to this app?
- How could you be best supported to incorporate Power Up into your clinical work?
- How could Power Up be improved?

Shared Decision Making

- What does shared decision making mean to you?
- When making decisions about the support young people receive in CAMHS, who do you think should be involved?
- Can you give an example of where you have used shared decision making?
  - How did you/ the young person find this experience?
  - What was good/ bad about this experience?
- Can you give an example of where you have not used shared decision making?
  - How did you/ the young person find this experience?
  - What was good/ bad about this experience?

Power Up topic guide for Parents/ Carers interviews and focus groups

Introduction + Aims

- Power Up project – Phase 1 + 2 – timelines
- Description of app
    - Support YP to get their voice heard in CAMHS
    - Space for them to record what's going on for them, and their experience of therapy in their own words (what they enter into the app cannot be digitally shared)
    - Video, audio, text, photo
    - Step by step plans, diary entries, questions, decision tool
    - Record sessions and flag things to talk about in session
    - My library
    - In sessions and between sessions
- Aims of today
    - Some feedback from you on the design and usability of the app
    - Some feedback on how the app could support the therapeutic work you do.
    - Some thoughts on how best to integrate the app into therapy.

Tools (Blind Test first)

- Open up the Power Up app and have a look. Read some of the descriptions of the tools as you open them and start having a play. Add some entries as if you were a user of this app.
- Talk me through your thoughts as you are looking at [Insert Tool name]
    - What do you like/ not like about what Power Up does and how it looks?
    - Is it clear what the tool is for?
    - Is the tool easy to use?

Use of Power Up

- How could Power Up have an impact on young people's experience of CAMHS?
- Which tools on the app do you think you would use the most/ least useful to your child?
- How might Power Up be useful/ not useful to your child?

- Can you give me an example of a time your child might want to use Power Up?
- If someone had given your child 'Power Up' before you started attending CAMHS, would they have found it useful? Why?

- Who/ what would you need to help you to support your child to use Power Up?
- Does anything worry you about your child using Power Up?

- How could Power Up be made better/ more useful for your child?

Shared Decision Making

- What does shared decision making mean to you?
- When decisions are being made about your child's care in CAMHS, who do you think should be involved?

- Can you give me an example of a time when shared decision making has happened during your child's care in CAMHS?
    - How did you/ your child find this experience?
    - What did was good/ bad about this experience?
- Can you give me an example of a time when shared decision making has not happened during your child's care in CAMHS?
    - How did you/ your child find this experience?
    - What did was good/ bad about this experience?

Power Up topic guide for Young Person interviews and focus groups

Introduction + Aims

- Power Up project – Phase 1 + 2 – timelines

- Description of app
    - Support YP to get their voice heard in CAMHS
    - Space for them to record what's going on for them, and their experience of therapy in their own words (what they enter into the app cannot be digitally shared)
    - Video, audio, text, photo
    - Step by step plans, diary entries, questions, decision tool
    - Record sessions and flag things to talk about in session
    - My library
    - In sessions and between sessions
- Aims of today
    - Some feedback from you on the design and usability of the app
    - Some feedback on how the app could support the therapeutic work you do.
    - Some thoughts on how best to integrate the app into therapy.

Tools (Blind Test first)

- Open up the Power Up app and have a look. Read some of the descriptions of the tools as you open them and start having a play. Add some entries as if you were a user of this app.
- Talk me through your thoughts as you are looking at [Insert Tool name]
    - What do you like/ not like about what Power Up does and how it looks?
    - Is it clear what the tool is for?
    - Is the tool easy to use?

Use of Power Up

- How could Power Up have an impact on young people's experience of CAMHS?
- Which tools on the app do you think you would use the most/ least useful to you?
- How might Power Up be useful/ not useful to you?

- Can you give me an example of a time you might want to use Power Up?
- If someone had given you 'Power up' when you started attending [Insert name of service], would you have found it useful? Why?

- Who/ what would you need to help you to use Power Up?
- Does anything worry you about using Power Up?

- How could Power Up be made better/ more useful for you?

Shared Decision Making

- What does shared decision making mean to you?
- When decisions about being made about your care in CAMHS, who do you think should be involved?
- Can you give me an example of a time when shared decision making has happened in your CAMHS sessions?

    - How did you find this experience?
    - What did was good/ bad about this experience?
- Can you give me an example of a time when shared decision making has not happened in your CAMHS sessions?
    - How did you find this experience?
    - What did was good/ bad about this experience?
